# Diet and Physical Activity for the Prevention of Noncommunicable Diseases in Low- and Middle-Income Countries: A Systematic Policy Review

**DOI:** 10.1371/journal.pmed.1001465

**Published:** 2013-06-11

**Authors:** Carl Lachat, Stephen Otchere, Dominique Roberfroid, Abubakari Abdulai, Florencia Maria Aguirre Seret, Jelena Milesevic, Godfrey Xuereb, Vanessa Candeias, Patrick Kolsteren

**Affiliations:** 1 Nutrition and Child Health Unit, Institute of Tropical Medicine, Antwerp, Belgium; 2 Department of Food Safety and Food Quality, Faculty of Bioscience Engineering, Ghent University, Ghent, Belgium; 3 Community Nutrition Department, School of Medicine and Health Sciences, University for Development Studies, Tamale, Ghana; 4 International Diabetes Federation, Brussels, Belgium; 5 Centre of Research Excellence in Nutrition and Metabolism, Institute for Medical Research, University of Belgrade, Belgrade, Serbia; 6 Global Strategy on Diet, Physical Activity and Health, Surveillance and Population-Based Prevention Unit, Department of Chronic Diseases and Health Promotion, World Health Organization, Geneva, Switzerland; The University of Queensland, Australia

## Abstract

**Background:**

Diet-related noncommunicable diseases (NCDs) are increasing rapidly in low- and middle-income countries (LMICs) and constitute a leading cause of mortality. Although a call for global action has been resonating for years, the progress in national policy development in LMICs has not been assessed. This review of strategies to prevent NCDs in LMICs provides a benchmark against which policy response can be tracked over time.

**Methods and Findings:**

We reviewed how government policies in LMICs outline actions that address salt consumption, fat consumption, fruit and vegetable intake, or physical activity. A structured content analysis of national nutrition, NCDs, and health policies published between 1 January 2004 and 1 January 2013 by 140 LMIC members of the World Health Organization (WHO) was carried out. We assessed availability of policies in 83% (116/140) of the countries. NCD strategies were found in 47% (54/116) of LMICs reviewed, but only a minority proposed actions to promote healthier diets and physical activity. The coverage of policies that specifically targeted at least one of the risk factors reviewed was lower in Africa, Europe, the Americas, and the Eastern Mediterranean compared to the other two World Health Organization regions, South-East Asia and Western Pacific. Of the countries reviewed, only 12% (14/116) proposed a policy that addressed all four risk factors, and 25% (29/116) addressed only one of the risk factors reviewed. Strategies targeting the private sector were less frequently encountered than strategies targeting the general public or policy makers.

**Conclusions:**

This review indicates the disconnection between the burden of NCDs and national policy responses in LMICs. Policy makers urgently need to develop comprehensive and multi-stakeholder policies to improve dietary quality and physical activity.

*Please see later in the article for the Editors' Summary*

## Abstract 2

Carl Lachat and colleagues evaluate policies in low- and middle-income countries addressing salt and fat consumption, fruit and vegetable intake, and physical activity, key risk factors for non-communicable diseases.

*Please see later in the article for the Editors' Summary*

## Editors' Summary

**Background:**

Noncommunicable diseases (NCDs)—chronic medical conditions including cardiovascular diseases (heart disease and stroke), diabetes, cancer, and chronic respiratory diseases (chronic obstructive pulmonary disease and asthma)—are responsible for two-thirds of the world's deaths. Nearly 80% of NCD deaths, close to 30 million per year, occur in low- and middle-income countries (LMICs), where they are also rising most rapidly. Diet and lifestyle (including smoking, lack of exercise, and harmful alcohol consumption) influence a person's risk of developing an NCD and of dying from it. Because they can be modified, these risk factors have been at the center of strategies to combat NCDs. In 2004, the World Health Organization (WHO) adopted the Global Strategy on Diet, Physical Activity and Health. For diet, it recommended that individuals achieve energy balance and a healthy weight; limit energy intake from total fats and shift fat consumption away from saturated fats to unsaturated fats and towards the elimination of trans-fatty acids; increase consumption of fruits, vegetables, legumes, whole grains, and nuts; limit the intake of free sugars; and limit salt consumption from all sources and ensure that salt is iodized. For physical activity, it recommended at least 30 minutes of regular, moderate-intensity physical activity on most days throughout a person's life.

**Why Was This Study Done?:**

By signing onto the Global Strategy in 2004, WHO member countries agreed to implement it with high priority. A first step of implementation is usually the development of local policies. Consequently, one of the four objectives of the WHO Global Strategy is “to encourage the development, strengthening and implementation of global, regional, national and community policies and action plans to improve diets and increase physical activity.” Along the same lines, in 2011 the United Nations held a high-level meeting in which the need to accelerate the policy response to the NCD epidemic was emphasized. This study was done to assess the existing national policies on NCD prevention in LMICs. Specifically, the researchers examined how well those policies matched the WHO recommendations for intake of salt, fat, and fruits and vegetables, as well as the recommendations for physical activity.

**What Did the Researchers Do and Find?:**

The researchers searched the Internet (including websites of relevant ministries and departments) for all publicly available national policies related to diet, nutrition, NCDs, and health from all 140 WHO member countries classified as LMICs by the World Bank in 2011. For countries for which the search did not turn up policies, the researchers sent e-mail requests to the relevant national authorities, to the regional WHO offices, and to personal contacts. All documents dated from 1 January 2004 to 1 January 2013 that included national objectives and guidelines for action regarding diet, physical exercise, NCD prevention, or a combination of the three, were analyzed in detail.

Most of the policies obtained were not easy to find and access. For 24 countries, particularly in the Eastern Mediterranean, the researchers eventually gave up, unable to establish whether relevant national policies existed. Of the remaining 116 countries, 29 countries had no relevant policies, and another 30 had policies that failed to mention specifically any of the diet-related risk factors included in the analysis. Fifty-four of the 116 countries had NCD policies that addressed at least one of the risk factors. Thirty-six national policy documents contained strategies to increase fruit and vegetable intake, 20 addressed dietary fat consumption, 23 aimed to limit salt intake, and 35 had specific actions to promote physical activity. Only 14 countries, including Jamaica, the Philippines, Iran, and Mongolia, had policies that addressed all four risk factors. The policies of 27 countries mentioned only one of the four risk factors.

Policies primarily targeted consumers and government agencies and failed to address the roles of the business community or civil society. Consistent with this, most were missing plans, mechanisms, and incentives to drive collaborations between the different stakeholders.

**What Do These Findings Mean?:**

More than eight years after the WHO Global Strategy was agreed upon, only a minority of the LMICs included in this analysis have comprehensive policies in place. Developing policies and making them widely accessible is a likely early step toward specific implementation and actions to prevent NCDs. These results therefore suggest that not enough emphasis is placed on NCD prevention in these countries through actions that have been proven to reduce known risk factors. That said, the more important question is what countries are actually doing to combat NCDs, something not directly addressed by this analysis.

In richer countries, NCDs have for decades been the leading cause of sickness and death, and the fact that public health strategies need to emphasize NCD prevention is now widely recognized. LMICs not only have more limited resources, they also continue to carry a large burden from infectious diseases. It is therefore not surprising that shifting resources towards NCD prevention is a difficult process, even if the human cost of these diseases is massive and increasing. That only about 3% of global health aid is aimed at NCD prevention does not help the situation.

The authors argue that one step toward improving the situation is better sharing of best practices and what works and what doesn't in policy development. They suggest that an open-access repository like one that exists for Europe could improve the situation. They offer to organize, host, and curate such a resource under the auspices of WHO, starting with the policies retrieved for this study, and they invite submission of additional policies and updates.

**Additional Information:**

Please access these websites via the online version of this summary at http://dx.doi.org/10.1371/journal.pmed.1001465.

- This study is further discussed in a *PLOS Medicine* Perspective by Stuckler and Basu
- The WHO website on diet and physical activity contains links to various documents, including a diet and physical activity implementation toolbox that contains links to the 2004 Global Strategy document and a Framework to Monitor and Evaluate Implementation
- There is a 2011 WHO primer on NCDs entitled Prioritizing a Preventable Epidemic
- A recent *PLOS Medicine* editorial and call for papers addressing the global disparities in the burden from NCDs
- A PLOS Blogs post entitled Politics and Global HealthAre We Missing the Obvious? and associated comments discuss the state of the fight against NCDs in early 2013
- The NCD Alliance was founded by the Union for International Cancer Control, the International Diabetes Federation, the World Heart Federation, and the International Union Against Tuberculosis and Lung Disease; its mission is to combat the NCD epidemic by putting health at the center of all policies
- The WHO European Database on Nutrition, Obesity and Physical Activity (NOPA) contains national and subnational surveillance data, policy documents, actions to implement policy, and examples of good practice in programs and interventions for the WHO European member states

## Introduction

Noncommunicable diseases (NCDs) are the leading cause of death globally. Of the 57 million global deaths in 2008, 36 million (63%) were due to NCDs, principally cardiovascular diseases, diabetes, cancers, and chronic respiratory diseases [1]. Mortality and morbidity data reveal the growing and disproportional impact of the epidemic in low- and middle-income countries (LMICs). Nearly 80% of the yearly NCD deaths—equivalent to 29 million people—are estimated to occur in LMICs. Without effective prevention and control, an estimated 41 million people in LMICs will die from NCDs by 2015 [2]. NCDs will evolve into a staggering economic burden over the next two decades [3].

Poor dietary quality (in particular, high salt intake, high saturated and trans-fatty acid intake, and low fruit and vegetable consumption) and insufficient physical activity are key risk factors for NCD development [4] and mortality worldwide [5], and are considered priority areas for international action [6]. The mean salt intake in most LMICs exceeds the recommended maximum intake [7]. Reducing salt intake to about 6 g/d could prevent annually about 2.5 million deaths globally [8]–[11], and a 15% reduction of salt intake over a decade in LMICs could forestall 3.1 million deaths [11],[12]. Fruit and vegetable intake is inadequate [13], and this situation contributes to 2.7 million NCD-related deaths per year. Despite evidence indicating that proper levels of physical activity are associated with a 30% reduction in the risk of ischemic heart disease, a 27% reduction in the risk of diabetes, and a 21%–25% reduction in the risk of breast and colon cancer [14],[15], approximately 3.2 million deaths each year are attributable to insufficient physical activity [6]. Physical inactivity is increasingly becoming prevalent in LMICs and already constitutes one of the leading causes of mortality [16]. There is also concern about excess intake of saturated and transfatty acids in LMICs, although large regional differences are observed [17],[18].

Preventing NCDs is not impossible [19]. Cecchini and colleagues analyzed population-based strategies to prevent NCDs in a number of LMICs with a high burden of NCDs [4]. Health information and communication strategies, fiscal measures, and regulatory measures for marketing or provision of nutrition information to children that promotes healthy eating and physical activity were found to yield substantial and cost-effective health gains, in particular in LMICs [4]. In addition, these interventions were found to be particularly effective when delivered as a multi-intervention package. Hence, it is crucial to translate the available evidence into sustainable policies in LMICs [6].

In May 2004, all WHO member states endorsed the Global Strategy on Diet, Physical Activity and Health, aiming to address NCDs through diet and physical activity [20]. Recently, a United Nations high-level meeting convened to discuss measures to prevent and control the global NCD epidemic and stressed the need to accelerate the policy response to it [21]. Monitoring this international commitment is important and can be achieved by systematic policy reviews. Previous policy reviews [22],[23], however, provided only a partial view of efforts undertaken to address NCDs, as they relied on survey data and did not consider the actual content of the policies. As policy documents are the culmination of existing social processes, they reflect the views of various stakeholders and are considered to be a reliable account of prevailing policy paradigms in a country [24]. We carried out a stocktaking exercise on national policy actions for NCD prevention in LMICs, and assessed the extent to which these address critical risk factors for NCDs, i.e., salt, fat, and fruit and vegetable intake, and physical inactivity. We focused on the existence and content of policies for the prevention of NCDs, not on their actual implementation.

## Methods

### Collection of Policy Documents

We searched the Internet (key words [“Nutrition” OR “NCD”] AND [“Policy” OR “Strategies” OR “Actions”]) for all publicly available national policies related to diet, nutrition, NCDs, and health in the countries classified as LMICs by the World Bank in 2011 [25]. We also searched the websites of the national ministries involved in nutrition or NCD prevention (i.e., ministries of health, sports, welfare, social affairs, or agriculture) and government portals as well as national nutrition societies. For those countries for which no policy was retrieved through the web search, an e-mail request stating the purpose of the study was sent to the respective bodies. A similar e-mail request was also sent to the WHO Regional Offices and to personal contacts of the research team. When no reply was obtained after repeated contact attempts and no reference to the existence of relevant policy documents was found during our Internet search, we classified the country as one for which we were unable to assess availability of policies. In addition to our search, we used the policy database of the WHO Regional Office for Europe [26] to assess policy availability.

### Screening and Selection of Documents

The following inclusion criteria were used to include the policies in the analysis: (i) the policy is from a country classified as LMIC according to 2011 World Bank classification [25], (ii) the policy is officially approved by the national government, (iii) the policy is a publicly available document, published between 1 January 2004 and 1 January 2013, and (iv) the policy relates directly or indirectly to prevention of NCDs (Text S1). We report our findings as a systematic policy review (Text S2). Because we present our results by WHO region, we also excluded countries that were not official member states of WHO in 2011. There was no language restriction. The definitions of “policy,” “action plan,” and “program” vary broadly among the national documents. For the purpose of the present review, a broad definition of policy was used, and all national documents that included the national objectives and guidelines for action in the domain of diet and/or physical activity and/or prevention of NCDs were included. No document was excluded based on its title (e.g., “plan” versus “policy” versus “strategy”).

### Data Analysis

Structured content analysis was conducted by coding the documents in NVivo 8 (QSR International). The documents were coded independently by two researchers to minimize bias induced by subjective coding. The coded documents were compared, and if coding agreement was <99% (as assessed using Kappa test agreement in NVivo), the coded text was manually reviewed for inconsistencies.

**Figure 1:**
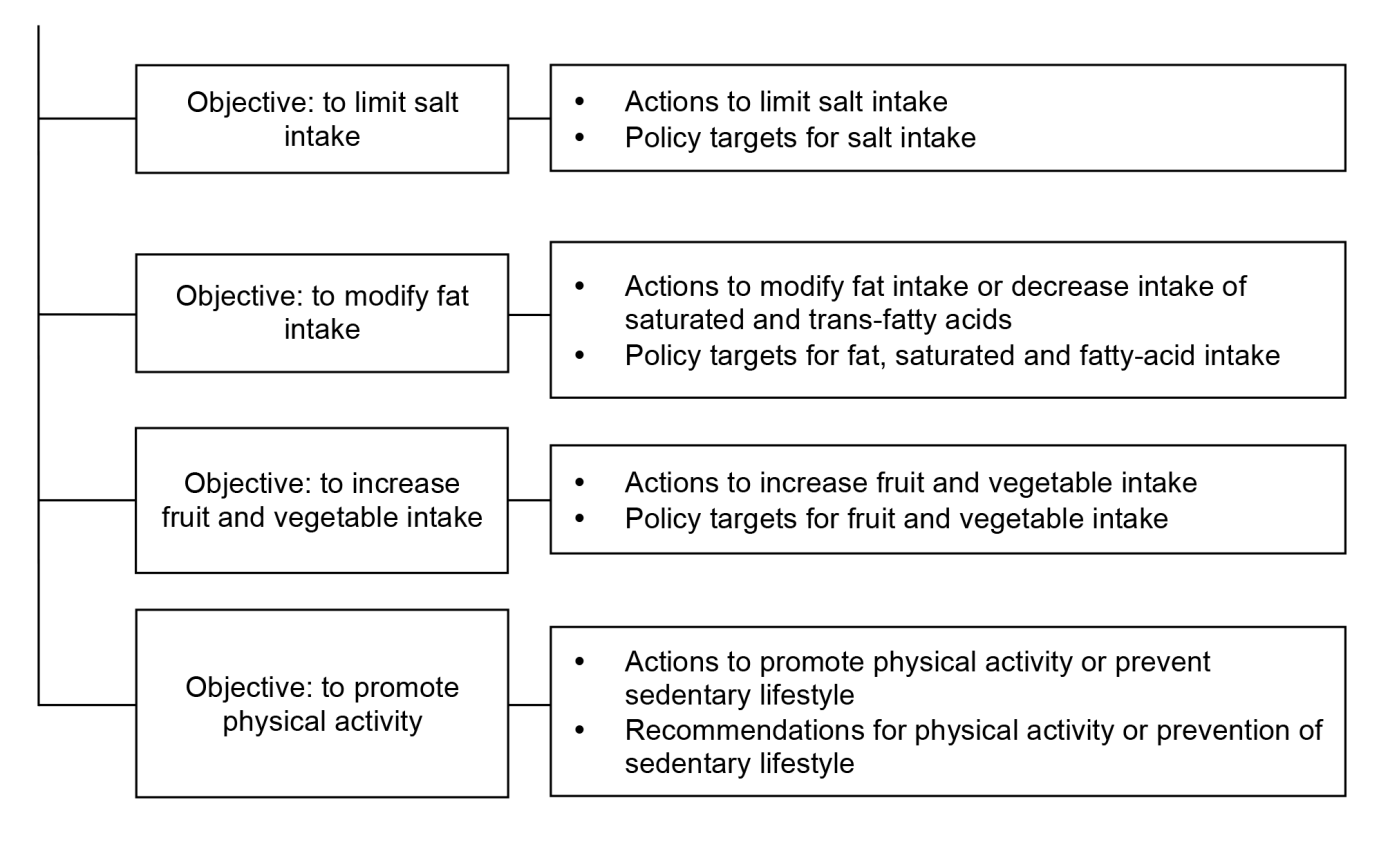
Coding tree for policy actions analyzed in the documents.

We coded all text that explicitly referred to actions aiming to (i) limit salt, (ii) modify fat intake, (iii) increase fruit and vegetable intake, or (iv) promote physical activity. Although we acknowledge that it is particularly the shift of fat consumption from saturated fats to unsaturated fats and the elimination of dietary trans-fatty acids that are critical for the prevention of NCDs, we extracted all strategies relating to dietary fat intake, such as reduction of total fat intake. The key words for coding were structured as a coding tree (Figure 1). A query was constructed for each topic in NVivo to extract all relevant text electronically. We present the results by the principle target audience of the actions, grouped into three categories: (i) general public and consumers, (ii) government, and (iii) private sector.

## Results

### Description of Policies

Out of the 144 countries classified by the World Bank as LMICs, four countries (Mayotte, West Bank and Gaza, the Republic of Kosovo, and American Samoa) were not official member states of WHO in 2011 and were excluded from the review. We note, however, the availability of a nutrition policy for West Bank and Gaza [27].

**Figure 2:**
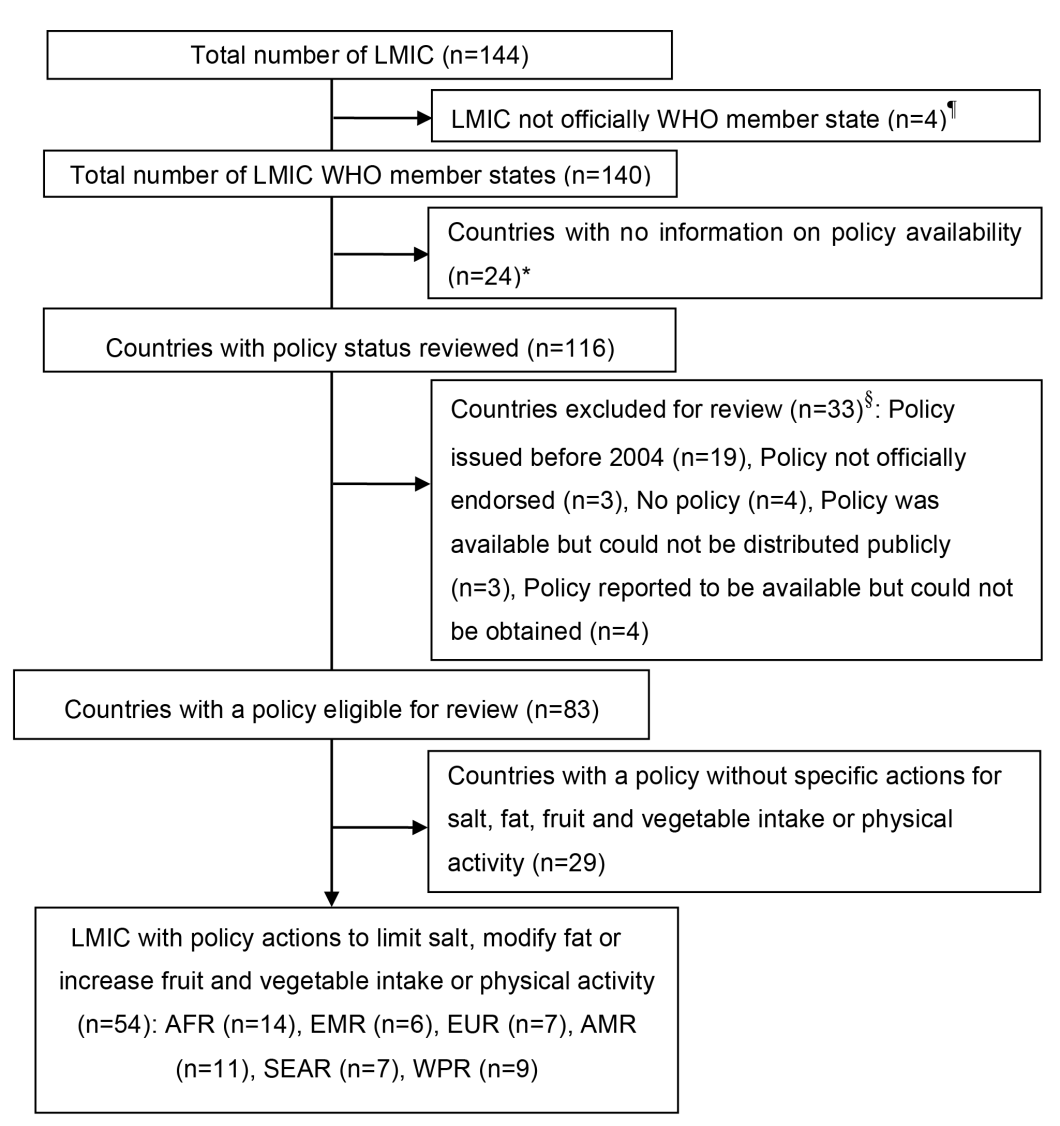
Selection process of nutrition, noncommunicable diseases, and health policies from low- and middle- income countries. The WHO classification of regions and allocation of countries was used. AFR, African Region; AMR, Region of the Americas; EMR, Eastern Mediterranean Region; EUR, European Region; SEAR, South-East Asia Region; WPR, Western Pacific Region. ^¶^Mayotte, West Bank and Gaza, the Republic of Kosovo, and American Samoa. *Antigua and Barbuda, Egypt, Dominica, Democratic People's Republic of Korea, Sao Tome and Principe, Dominican Republic, Micronesia, Gabon, Tonga, Kyrgyzstan, Lebanon, Libya, Algeria, Iraq, Lithuania, Palau, Marshall Islands, Uzbekistan, Yemen, Romania, Saint Kitts and Nevis, Syrian Arab Republic, Turkmenistan, and Comoros. ^§^Policy issued before 2004: Belize, Venezuela, Bosnia and Herzegovina, Eritrea, Lesotho, Papua New Guinea, Albania, Armenia, Burundi, Ecuador, El Salvador, Kiribati, Namibia, Sierra Leone, Gambia, Zimbabwe, Somalia, United Republic of Tanzania, and Vanuatu; policy not officially endorsed: Democratic Republic of the Congo, Senegal, and Tuvalu; no policy : Chad, Congo, South Africa, and Tajikistan; policy was available but could not be publically distributed: Central African Republic, Cameroon, and Tunisia; policy reported to be available [26] but could not be obtained: Azerbaijan, Belarus, Kazakhstan, and Ukraine.

Of the 140 LMICs found in the six WHO regions (Africa, Europe, the Americas, South-East Asia, the Eastern Mediterranean, and the Western Pacific), we found information on the availability of policies for 83% (116/140) countries (Figure 2; Table S1). We were unable to assess the availability of policy documents for 24 countries, and in particular in the Eastern Mediterranean Region 40% (6 out of 15 countries in the region). In the European, African, Western Pacific, and South-East Asian Regions and the Region of the Americas, this proportion was 24% (5/21), 9% (4/45), 22% (4/18), 9% (1/11), and 13% (4/30), respectively. In total, 33 countries were excluded from review as they had no policy (*n = *4), a policy published before 2004 (*n = *19), or a policy document that was not officially endorsed (*n = *3) or could not be circulated publicly (*n = *3). In an additional four countries, a policy was reported to be available [26], but the full document could not be obtained.

**Figure 3:**
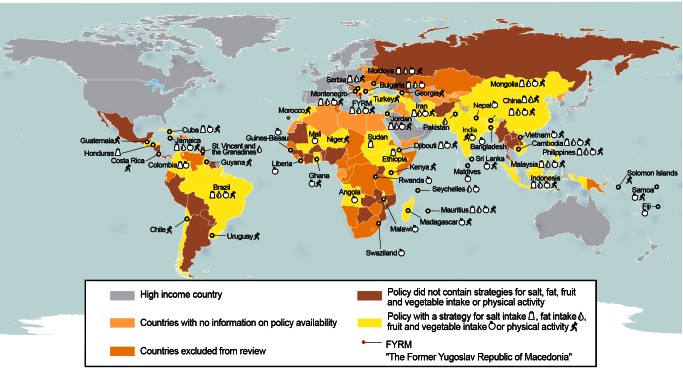
Atlas of availability of national actions to limit salt or fat intake or increase fruit and vegetable intake or physical activity. Geographic boundaries from the United Nations Cartographic Section were used [105].

For 29 countries, the policy document reviewed did not contain any of the NCD prevention strategies reviewed [28]–[56]. In 30 countries, policy strategies to improve dietary quality did not specify actions for any of the dietary risk factors reviewed here [28],[29],[32]–[38],[40]–[52],[55]–[62]. In the countries reviewed, strategies that addressed intake of salt, fat, or fruits and vegetables or the promotion of physical activity were found in 47% (54/116) of policies. These policies had a main focus on food or nutrition (*n = *34), general health (*n = *11), and, to a lesser extent, the prevention of NCDs (*n = *9). In total, 36 countries had explicit actions in their policies to increase fruit and vegetable intake, 20 specified actions aimed to address dietary fat consumption, 23 specified actions to limit salt intake, and 35 specified actions to promote physical activity. Although generally low, the proportion of countries with a policy that targeted at least one risk factor was higher in South-East Asia and the Western Pacific than in Africa, Europe, the Americas, and the Eastern Mediterranean. Only 12% (14/116) of the LMICs reviewed (Bhutan, Jamaica, Mauritius, the Republic of Moldova, Malaysia, Indonesia, the Philippines, Cambodia, the former Yugoslav Republic of Macedonia, Jordan, Montenegro, Brazil, Iran, and Mongolia) proposed a package that addressed all four risk factors, and approximately 23% (27/116) of countries addressed only one of the risk factors (Figure 3).

### Policy Actions to Limit Salt Intake

Only 20% (23/116) of the countries reviewed specified strategies to limit dietary salt intake, and eight policies detailed national targets to limit salt intake (Table 1). A large majority (83%; 19/23) of the countries with salt reduction strategies outlined measures of education and awareness creation in the general public and consumers, in particular focused on food labeling and promotion of foods, snacks, and packaged seasonings with reduced salt content. Strategies targeted towards the private sector were observed in 30% (7/23) of the policies and mainly related to product reformulation. The actions targeted at the governments were essentially the establishment of fiscal measures, labeling, and development of standards for salt in food and market regulations. Of the 83 countries with a policy eligible for review, 43 contained specific measures for salt iodization, but only ten of these also mentioned the need to reduce or manage dietary salt intake.

**Table 1:** National policy actions and targets to limit salt intake in LMICs by WHO region and target group.

Target Group	African Region	Eastern Mediterranean Region	European Region	Region of the Americas	South-East Asia Region	Western Pacific Region
**General public and consumers**	**Mauritius:** Public education on food labeling and moderate salt intake	**Sudan:** Promote reduction in salt intake; **Djibouti:** Promote reduction of salt intake; **Iran:** Address poor dietary habits and reduce the consumption of salt; public education through TV regarding low salt consumption	**Bulgaria:** Enforce lower salt intake in school canteens; **Bulgaria and the Republic of Moldova:** Educate on salt intake reduction; **Serbia:** Increase intake of food with high ratio of potassium/sodium	**Jamaica and Cuba:** Public education to reduce salt intake	**Bhutan:** Public education on salt intake reduction, targeting school children; **Indonesia:** Public education on salt intake reduction targeting high-risk groups; promotion of low-salt eating habits	**Malaysia and Mongolia:** Public education on salt intake reduction; **Malaysia:** Encourage the public to use less salt and choose foods low in salt
**Government**	**Ethiopia:** Enforce food standards and salt legislation; **Mauritius:** Amend food regulations to include signpost labeling and enforce traffic light signpost labeling with reference to salt content	**Sudan:** Propose taxes on salty foods; **Jordan:** Develop national recommendations for the reduction of salt intake	**Bulgaria:** Introduce standards for upper limits of salt for some foods; introduce taxes and fiscal measures on high-salt foods; **Montenegro:** Development of guidelines for food production with lowered content of salt; **Serbia:** Mandatory labeling of sodium content and potassium/sodium ratio for food producers; harmonize recommendation for reduction of salt in processed food	**Jamaica:** Promote production and sale of foods with less salt and the consumption of products low in salt; **Honduras:** Strengthen food labeling policies for salt; **Brazil:** Agreement and partnership between the production sector and the public sector to prevent NCDs by promoting salt reduction, to reach the suggested national goals on reduction of salt; **Colombia:** Disseminate, monitor, and regulate the nutritional labeling of foods to control the amount of salt in processed foods	**Bhutan:** Establish guidelines to control marketing and advertisement of salty foods	**Mongolia:** Review and update legislative acts and standards to promote production, sale, and importation of low-salt foods; coordinate foreign trade policy to support decrease in salt intake; **Philippines:** Develop and implement health promotion activities for a healthy diet that limits salt intake from all sources; **Cambodia:** Incorporate nutrition messages related to low salt intake in the curriculum; develop standards for school and university vendors; **China:** Develop and promote healthy foods with low salt content
**Private sector**	**Mauritius:** Train food industry and stakeholders on enforcement of food labeling with reference to salt content	**Iran:** Improve nutrition in public places through policies for reduced salt in food industries and restaurants; sensitize food producers regarding the reduction of salt in food products	**Bulgaria:** Produce foods with reduced salt content; **FYRM:** Change formulation of foods to reduce salt content	**Brazil:** Regulate the nutritional composition of processed food; establish an agreement with the production sector and a partnership with the civil society to prevent NCDs and reduce salt in food; reduce salt in industrialized food by 10% per year on voluntary basis	**Indonesia:** Collaborate with stakeholders for reduction of salt in processed foods	**Mongolia:** Collaborate with stakeholders to reduce salt content of processed foods
**National salt intake target**	**Mauritius:** Reduce national average sodium intake to <5 g/d	NR	**Bulgaria, Montenegro, and FYRM:** Reduce salt intake to <5 g/d; **Serbia:** Limit intake of salt to <6 g/d	**Cuba:** Increase the proportion of people who do not add salt on the table to 95%; **Brazil:** Reduce average salt consumption	NR	**China:** Lower national average per capita intake of salt to <9 g

WHO classification of regions and countries was followed.

FYRM, the former Yugoslav Republic of Macedonia; NR, not reported.

### Policy Actions to Modify Fat Intake

Of the countries with strategies to modify fat intake, 65% (13/20) proposed strategies targeting the general public and consumers via public education and awareness creation (Table 2). The use of dietary guidelines and food labeling were specifically mentioned as means of public education on dietary fat intake reduction in the Mauritius [63], Bulgaria [64], Jamaica [65], and Bhutan [66]. Imposition of fiscal measures, collaboration with the food industry for product reformulation, and the establishment and enforcement of food standards were mentioned as the main actions to be implemented by the government. Only Mauritius [63], Bulgaria [64], the former Yugoslav Republic of Macedonia [67], Iran [68], and Mongolia [69] outlined specific strategies targeted towards the private sector. Intake of specific fatty acids, and in particular saturated fat (Mongolia [69], the former Yugoslav Republic of Macedonia [67], Jordan [70], Bhutan [66], Cambodia [71], Bulgaria [64], Seychelles [72], the Philippines [73], Montenegro [74], and Iran [68]) and trans-fatty acids (the former Yugoslav Republic of Macedonia [67], Bhutan [66], Bulgaria [64], Seychelles [72], the Philippines [73], Montenegro [74], Mauritius [63], Brazil [75], and Iran [68]), was addressed in ten and nine countries, respectively. Whereas Mauritius [63] proposed research into the safety of reused oils, others focused on the type of fat (Iran [68] and Cambodia [71]) or the number of times oil should be used (Seychelles [72]). Six countries (Mauritius [63], Bulgaria [64], the former Yugoslav Republic of Macedonia [67], Montenegro [74], Malaysia [76], and Viet Nam [77]) mentioned specific national fat intake targets (Table 2).

**Table 2:** National policy actions and targets to limit fat intake by WHO region and target group.

Target Group	African Region	Eastern Mediterranean Region	European Region	Region of the Americas	South-East Asia Region	Western Pacific Region
**General public and consumers**	**Mauritius:** Public education on fat intake reduction through dietary guidelines and food labeling; **Seychelles:** Public education on use of poly- and monounsaturated fats and reusing oils; oils used for frying should not be reused on more than two occasions, to reduce intake of trans-fatty acids	**Pakistan:** Initiate community-based behavior change communication strategies to control high fat intake; **Jordan:** Organize campaigns for the importance of low-fat food; **Iran:** Address poor dietary habits and reduce the consumption of fats and oils; promote traditional methods of food preparation and cooking e.g., use of liquid oil and discouraging food frying	**Bulgaria:** Promote products with low content of fats, saturated fatty acids, trans-fatty acids, and cholesterol; **Bulgaria and the Republic of Moldova:** Education on fat intake reduction	**Jamaica:** Market alternative products of high acceptability with lower fat content; labeling of products to permit informed choices by consumers; promote the consumption of products low in fat; **Saint Vincent and the Grenadines:** Public education on fat intake reduction	**Bhutan:** Public education on fat intake reduction targeting school children, dietary guidelines, food labeling, advertising, and marketing, to reduce trans-fatty acid and saturated fat intake; **Indonesia:** Public education on fat intake reduction; promotion of low-fat eating habits	**Malaysia and Mongolia:** Public education on fat intake reduction; reduce consumption of fried and conserved food; **Mongolia:** Provide intensive information, education, and communication activity towards reducing consumption of animal fat; **Malaysia:** Encourage people to minimize fat in food preparation and choose foods that are low in fat and cholesterol
**Government**	**Mauritius:** Healthy eating and good nutrition is included in the curriculum of basic schools to discourage the consumption of food items containing high levels of oils and fats; the Nutrition Taskforce to commission a study into the reuse of cooking oils and propose legislative measures to reduce trans-fatty acid intake; deep frying of foods in oils and/or fats will be discouraged and will be excluded from governmental food services and government functions	**Pakistan:** Develop policies and strategies to limit the production and access to ghee as a medium for cooking; **Jordan:** Develop national recommendations for total and saturated fat intake; **Iran:** Develop and publish educational materials regarding oil, its usage, and the disadvantages of saturated and trans-fatty acids for health personnel and other employees	**Bulgaria:** Introduction of standards for upper limits of fats, saturated fatty acids, and cholesterol for some foods; develop dietary guidelines to promote low-fat meat products and low-fat milk and dairy products; **Montenegro:** Development of guidelines for food production with lowered content of fat; **Serbia:** Harmonize recommendation for reduction of fat in processed food	**Brazil:** Set goals to reformulate processed food by reducing fat content; disseminate and monitor agreements and partnerships between the private sector and the civil society, to reach the suggested goals on reduction of trans fat	**Bhutan:** Establish guidelines to control marketing and advertisement of fatty foods, especially to children; increase tax on food items that are health harming; restrict fast food licenses	**Mongolia:** Improve control on nutrition quality and fat content of imported food products, and create legislative environment to promote healthy food products by taxation policy; update, approve, and implement food standards with reduced level of fat content, i.e., a reduction of animal fat consumption; promote healthy diet by reviewing and updating food standards in order to reduce fat intake; coordinate and monitor foreign trade policy in order to support consumption of low-fat-content food; **China:** Develop and promote healthy foods with low fat content; **Philippines:** Develop and implement health promotion activities for a healthy diet that limits energy intake from total fat and shifts saturated fat to unsaturated fat and towards the elimination of trans-fatty acids; **Cambodia:** Incorporate nutrition messages related to low saturated fat intake in the curriculum; develop standards for school and university vendors; study potential for subsidies on vegetable oils
**Private sector**	**Mauritius:** Train food industry and stakeholders on enforcement of food labeling with reference to fat content	**Iran:** Improve nutrition in public places through policies for reduced fat in food industries and restaurants; increase the production of low- and zero-fat products (i.e., dairy sector) in food industry	**Bulgaria:** Produce foods with low content of fats, saturated and trans-fatty acids, and cholesterol; **FYRM:** Change content of foods to reduce saturated fatty acids and trans-fatty acids	NR	NR	**Mongolia:** Encourage food industry and catering to produce and serve foods that decrease the consumption of animal fat
**National fat intake target**	**Mauritius:** Decrease the national consumption of oils and fats by 10% within 5 y	NR	**Bulgaria:** Reduce population fat intake to 30% of total energy; **FYRM:** Reduce saturated fat to <1%^a^ of total energy intake and trans-fatty acids to <1% of energy intake; **Montenegro:** Reduce intake of saturated fats to <10% and intake of trans-fatty acids to <1% of total energy intake	NR	NR	**Malaysia:** Decrease the proportion of people with dietary fat intake >30% of total calories, compared to the First Malaysian Food Consumption Survey; **Viet Nam:** Proportion of households with a diet with 14% of protein, 16% of lipids and 5–68% of carbohydrates is 50% by 2015 and 75% by 2020

WHO classification of regions and countries was followed. Four of the countries (Mayotte, West Bank and Gaza, the Republic of Kosovo, and American Samoa) classified as LMICs by the World Bank in 2011 [25] were not WHO member states in 2011.

a The policy document of FYRM reports that goals are in line with those of WHO [106]. The stated goal of <1% of total energy intake from saturated fat is therefore likely meant to be the WHO goal of 10%.

FYRM, the former Yugoslav Republic of Macedonia; NR, not reported.

### Policy Actions to Increase Fruit and Vegetable Intake

Compared to the other dietary risk factors reviewed, the objective of increasing fruit and vegetable consumption had the highest coverage: 31% (36/116) of the policies reviewed (Table S1). Promotion of school gardening, home gardening, and urban agriculture were the main actions to ensure availability and accessibility of fruit and vegetables (Table 3). The majority (75%; 27/36) of the policy documents with strategies for fruit and vegetable intake focused on public education and demonstrations to promote increased fruit and vegetable intake. Malaysia proposed the development of special recipe books in this regard [76]. Other strategies, as found in Sri Lanka [78] and Mongolia [69] for instance, targeted the catering services in educational and government institutions to ensure strict inclusion of fruits and vegetables in the meals. In all of the WHO regions, policy documents that addressed increasing fruit and vegetable consumption included the need to produce, store, and process local fruits and vegetables, and to educate populations to consume them. Policy measures outlining responsibilities for the private sector were less frequently encountered (28%; 10/36) than those detailing actions to be implemented by the government (53%; 19/36) or targeting the general public (75%; 27/36).

**Table 3:** National policy actions and targets to increase fruit and vegetable intake by WHO region and target group.

Target Group	African Region	Eastern Mediterranean Region	European Region	Region of the Americas	South-East Asia Region	Western Pacific Region
**General public**	**Liberia:** Diversify diet to improve nutritional quality, through promoting consumption of micronutrient-dense food and FV to complement staple foods; **Malawi:** Promote school gardens and cooking demonstrations in all public primary schools; develop urban agriculture and small gardens at homesteads and schools to produce FV and other nutritional produce; organize community-level demonstrations of preparation and consumption of locally available nutritious foods, such as indigenous FV and legumes to reach at least 5 million people; **Mali:** Promote consumption of FV in the population to diversify the agricultural production; **Swaziland:** Promote growing nutritious food crops such as FV by individuals and communities; increase public education and awareness about nutrition practices; develop urban agriculture and small gardens at homesteads and schools to produce FV and other produce; **Angola:** Increase consumption of foods that improve dietary patterns, such as FV, and of traditional foods; **Rwanda:** Promote consumption of FV at household level; **Guinea Bissau:** Promote production of vegetables to diversity crop production and increase the consumption of foods rich in micronutrients; **Ghana:** Promote FV production in household, school, and community gardens and institutions	**Djibouti:** Promote family orchards and the consumption of FV; **Jordan:** Organize campaigns for the importance of low-fat food such as FV; **Iran:** Address poor dietary habits and promote consumption of FV	**Bulgaria:** Stimulate and support local (rural and urban) production of FV, planting orchards, and the greenhouse production of vegetables; **Republic of Moldova:** Develop and popularize the national nutrition pyramid to reduce consumption of refined foods and increase consumption of FV; **Montenegro:** Support and offer simulative measures for production of FV and to ensure quality, availability, and supply	**Jamaica:** Promote an increased consumption of FV; promote consumption of FV without excess increase in body weight in the young child and adolescent population	**Bangladesh:** Promote homestead gardening, including FV farming, social forestry, livestock and backyard poultry, in homestead areas in flood-free years; **Bhutan:** Health promotion, community-based programs, and mainstreaming of control and prevention of NCDs in early learning centers, schools, universities, and monastic institutions to increase consumer demand for fruit; **Indonesia:** Promote the consumption of FV; assure food resilience on family and individual levels, with sufficient stock and access to food and balanced and safe nutrition, including FV commodities; increase socialization and advocacy for consumption of FV; **Sri Lanka:** Produce carotene-rich FV in homesteads; encourage nutritious breakfast and snack or fruit during the school break; guidelines for school canteens to provide low-cost, nutritious, clean, and wholesome food, including local fruit; encourage growth of carotene-rich FV in all educational institutions; **Nepal:** Promote home production gardens for fruit production; **India:** Promote fruits and leafy vegetables to diversify food for children and adolescents, especially girls; promotion of community- and household-level production of FV	**Fiji:** Encourage and support sustainable community-based healthy food production to increase consumption of FV; **Malaysia:** Promote dietary diversification to increase promotion activities for micronutrient-rich foods, including FV; **Mongolia:** Provide information, education, and communication activity towards acquiring healthy diet behavior among population by increasing FV intake; **Samoa:** Promote increased intake of FV, e.g., FV gardening; assist government women representatives in encouraging villagers to produce vegetable gardens for nutrition purposes and healthy diets; work with school canteens; **Viet Nam:** Promotion of daily consumption of vegetables; **Cambodia:** Offer homestead gardening and develop school vegetable gardens; encourage community leaders to develop local solutions such as community FV gardens
**Government**	**Liberia:** Expand production and marketing of nutritious vegetables to increase food availability and access through direct consumption, sales, and income; **Madagascar:** Improve and diversify food crop production in community nutrition sites, in particular in food-insecure zones, by promoting home gardens, fruit trees, etc.; **Malawi:** Facilitate economic empowerment to increase household and community access to food resources; set up a FV promotion initiative; increase the availability of FV at national level; **Mauritius:** Establish dietary recommendations for adults for the prevention of NCDs: 400 g of FV per day; launch FV consumption initiative to ensure integrated approach using different aspects, such as production availability; tea breaks at governmental functions will be replaced by health breaks using healthy alternatives such as fruits and nuts; **Swaziland:** Improve marketing of maize and FV; develop and promote sustainable production and processing of indigenous non-timber forest products, e.g., edible fruit; **Seychelles:** Develop guidelines and recommendations for fresh FV	**Jordan:** Develop national recommendations for the consumption of FV	**Bulgaria:** Stimulate and support the establishment of better conditions for production and adequate storage of local FV to supply communities during the whole year; assist in marketing; provision of seedlings, information, and free consultations; establish local networks for the distribution of the produced vegetables and plants; **FYRM:** Improve availability and access to FV through revised agricultural policies	**Colombia:** the compliance of regulation and nutrition labeling of food and promote consumption of FV (visible labels and other reinforcements); **Cuba:** Increase the availability of FV and follow the trend of consumer price index; **Brazil:** Encourage fiscal measures to reduce the price of healthy foods such as FV and increase their consumption	**Bhutan:** Develop policies encouraging rural population to increase production and availability of FV at affordable prices; develop policies and strategies to enhance production of FV and to ensure food security, especially for poor and marginalized groups; **Bangladesh:** Promote efficient use of available land, agricultural inputs, and water use for irrigation and for production of fruit; support appropriate research and agricultural loans; **Maldives:** Expand the agriculture sector to meet household and local demand for FV	**Malaysia:** Develop recipe books for vegetables to increase fiber intake; **Philippines:** Develop and implement health promotion activities for healthy diet and increased consumption of FV; **Cambodia:** Incorporate nutrition messages related to increased FV consumption in the curriculum; develop standards for school and university vendors; **Mongolia:** Coordinate and monitor foreign trade policy in order to support consumption of FV
**Private sector**	**Liberia:** Diversify food produced, including FV and livestock; **Mali:** Improve the transport of perishable foods such as FV; **Mauritius:** Promote FV in collaboration with other stakeholders to allow an integrated approach with production, availability, and promotion	NR	**Bulgaria:** Mandatory inclusion of fresh FV and milk or dairy products in the diet of children in kindergartens and crèches; strict control on provision at school cafeterias of fresh, seasonal FV, milk, yogurt, and milk/yogurt-based drinks	**Brazil:** Stimulate consumption of healthy food, such as FV	**Bhutan:** Encourage farmers to consume and grow FV using organic fertilizers and enhance income generation through sale of such products; **Sri Lanka:** Encourage catering sector to follow dietary guidelines and to offer foods such as kurakkan and bread fruit; **Indonesia:** Increase the consumption of vegetables by 4.5% and fruits by 5% per year	**Fiji:** Encourage proper processing of local food and FV; **Mongolia:** Encourage food industry and catering to produce and serve foods that increase FV consumption
**Policy targets**	**Mauritius:** Increase the consumption of FV by 2-fold (≥400 g/d); increase availability of FV, preferably by local production to ensure freshness of produce; **Seychelles:** Every meal should contain at least 100 g of FV; FV should be served in an appealing and easy-to-eat way; as much as possible, vegetables should be served free of added oil or fat; **Angola:** Intensify actions that promote production of foods rich in vitamins, particularly traditional vegetables, animal origin products, and revenue-generating produce; **Malawi:** Promote backyard gardens and planting of fruit trees; promote consumption of adequate food in quality and quantity to meet the nutritional needs for rural and urban households, with special emphasis on vulnerable groups and low-income households; promote consumption and availability of FV in the guidelines	NR	**Bulgaria:** Increase the consumption of FV in winter and spring up to 400 g daily; ensure proper storage of FV in winter; construct local storehouses for FV and provide access to them for small farms; **Republic of Moldova:** Promote the consumption of FV and other foods; **FYRM:** Average intake of at least 500 g of FV daily; **Montenegro:** Increase FV availability; increase consumption to >400 g/d	**Cuba:** Increase the proportion of the population consuming at least 200 g of FV by 40% and consuming at least three portions of vegetables per day or 300 g by 50%; **Jamaica:** A 20% increase in consumption of FV by December 2008; **Brazil:** Increase FV consumption	**Indonesia:** Increase consumption of FV daily; **Bangladesh:** Increase production of noncereal crops (FV, oilseeds, pulses)	**Fiji:** Increase intake of FV and promote healthy and safe diets to reduce NCDs; **Malaysia:** Diversify diets to increase the consumption of micronutrient-rich foods including FV; increase proportion of people consuming FV; **Mongolia and Samoa:** Increase the consumption of FV by households; **Philippines:** Increase per capita total vegetable consumption from 111 g/d to 133 g/d

WHO classification of regions and countries was followed. Four of the countries (Mayotte, West Bank and Gaza, the Republic of Kosovo, and American Samoa) classified as LMICs by the World Bank in 2011 [25] were not WHO member states in 2011.

FV, fruits and vegetables; FYRM, the former Yugoslav Republic of Macedonia; NR, not reported.

### Policy Actions to Increase Physical Activity and Address Sedentary Lifestyle

Public education and sensitization were the main strategies to promote physical activity in the policies (Table 4). Whereas countries such as Morocco [59], Mongolia [69], and Mauritius [63] targeted educational institutions, others, such as Bhutan [66], Guyana [79], and Malaysia [76], focused on workplaces. Samoa [80], the Niger [61], Indonesia [81], India [82], and Cambodia [71] targeted the community at large. Nine countries (Kenya [60], Morocco [59], Cuba [83], Uruguay [58], Jamaica [65], Brazil [75], Malaysia [76], the Philippines [73], and China [84]) proposed national policy targets for physical activity (Table 4).

**Table 4:** National policy actions and targets to promote physical activity by WHO region and target group.

Target Group	African Region	Eastern Mediterranean Region	European Region	Region of the Americas	South-East Asia Region	Western Pacific Region
**General public and consumers**	**Ghana:** Encourage regular exercise^a^; **Mauritius:** Emphasize maintaining a healthy weight by undertaking adequate PA in the dietary guidelines; **Niger:** Promote healthy lifestyles in families and communities so that sport and relaxation are widespread in neighborhoods; **Kenya:** Train health workers on PA; organize sensitization meeting on PA in counties	**Djibouti:** Promote PA and the creation of playgrounds; **Morocco:** Promote PA in schools and universities	**Georgia:** Make school sports facilities available for public use; provide nationwide evidence-based advocacy on the health, social, and economic benefits of PA; create an environment conducive for PA; urban planning policy choices should include: plan for stadia, safe walking routes, safe cycling routes, shelters from poor weather, and recreational facilities; **Republic of Moldova:** Population-wide communication to promote PA, including in elderly and sedentary population; **Turkey:** Provide correct information to the public by written and visual media on active life and obesity; **FYRM:** Recommendations for proper nutrition are always followed by recommendations for PA; **Montenegro:** Support local government in designing models for PA facilities and building of safe roads for bikers and pedestrians in settlements; conduct educative programs on the importance of PA in school curriculum; **Serbia:** Promote and implement PA in everyday life in population	**Chile:** Promote PA at workplaces, disseminate PA guidelines to the population, preschool and school children; **Costa Rica:** Promote healthy lifestyles and PA and recreation; **Cuba:** Promote intersectoral participation in systematic PA at the workplace and intersectoral participation in systematic PA in the general population; **Guyana:** Promote PA in communities and schools; **Uruguay:** Develop guidelines for PA and lifestyle for the general population; **Guatemala:** Apply strategies and measures to promote good health that include PA, especially in the workplace and schools; multisectoral workshops for the formation of local and national PA networks; **Brazil:** Promote active aging, e.g., through private health plans, and encourage the elderly to engage in regular PA; encourage PA in children on an everyday basis and throughout life; promote leisure PA and healthy lifestyle for children and young people; guidelines promote providing two physical education classes a week at schools; communication and education campaign to promote health through PA	**Bhutan:** Advocate at the population level for PA in the workplace; encourage walking and regular physical exercise, with a focus on the urban and more sedentary population; increase PA at the population level by enhancing understanding among the general public that more PA leads to better health; **Indonesia:** Increase understanding of the benefit of PA; increase PA of people through increase of promotion; increase in provisions of means and facilities of sports and open space, in the frame of creating awareness at all levels of society; **Sri Lanka:** Create of awareness on PA; promoting greater PA among school children and adults will also reduce the risk of chronic degenerative diseases; provision of facilities for outdoor recreation and making all roads safe for pedestrians and cyclists; **India:** Physical education to be built into the school system; creation of sports infrastructure at grassroots level in rural and urban areas; revision of the sports policy and action plan and services; involvement of corporate sector	**Mongolia:** Introduce basic knowledge about PA into curriculum of secondary schools; population-wide promotion of PA; **Samoa:** PA is one of the four high-risk areas identified to focus on through health promotion programs; emphasis on community groups, women's communities, and government workers to support healthy lifestyle, including PA; promote PA in elderly homes; **Solomon Islands:** Assist individuals who have been disabled by disease, traumatic injury, or other causes to achieve their maximum potential in terms of PA; promote maintenance of body weight by balancing food intake with regular PA; **Malaysia:** Promote physical fitness activities for the general population at the workplace; **Cambodia:** Public awareness of healthy lifestyles, and lack of PA as a risk factor, in particular in women; encourage community leaders to develop local solutions such as walking groups or green space; **China:** Ensure that primary and secondary students participate in at least 1 h of physical exercise activities during the school day; the communities shall actively promote the working model of healthy lifestyle instructors and social sports instructors
**Government**	**Ghana:** Make PA education mandatory in all schools^a^; **Mauritius:** Ministries of health and finance will collaborate to have a strong focus on PA among the elderly; **Madagascar:** Develop a policy for the prevention of NCDs with PA recommendations	**Morocco:** Advocate for public space and an environment conducive for PA; **Jordan:** Develop a national strategy for the promotion of exercise and PA: develop multisectoral committee for PA; **Iran:** Increase PA to prevent and control overweight and obesity in students	**Georgia:** Develop local legislation and policy to support PA; health sector to take the leading role in making policy decisions by developing action-oriented networks with other relevant sectors and stakeholders on PA; allocate a proportion of sport funds to promoting PA; **Republic of Moldova:** Extend urban public green space and special grounds for PA for the population; revival of regular short breaks in schools and worksites and encouragement of PA through curricula and support; **Turkey:** Establish provincial coordination centers for Obesity Prevention, Nutrition and Active Life Board in 81 provinces; mainstream obesity-fighting strategies in national health strategies and policies; prepare national PA guidelines; improve education program related to PA in the educational system; improve the environment for PA in the educational system; formation of sports facilities and recreational areas within the budget possibilities, with the leadership of local administrations, for making the PA in the public widespread; development of PA applications that can be easily applied inside the house; **FYRM:** Increase possibility for PA through integration of PA in everyday life, e.g., in kindergartens, schools, and worksites; support for local authorities for recreational infrastructure and elimination of barriers for PA transport; **Montenegro:** Local governments provide conditions for development of infrastructure and facilities for PA: swimming pools, playgrounds, parks, and cycling and walking paths; conduct activities toward development of conditions for cycling and walking in traffic; development of programs for PA in kindergartens, schools, and universities; awareness creation in media; **Serbia:** Perform moderate PA according to national guidelines; promote PA in children, adolescents, adults, elderly individuals, healthy individuals, and patients with cardiovascular disease; upgrade programs for PA in school curriculum; educate medical and PA professionals on PA for patients with cardiovascular disease; develop and enforce the collaboration between government and NGOs in implementation of PA recommendations; government and NGO campaign “Sport for Everyone”	**Chile:** Develop population PA guidelines*;* **Guyana:** Introduce PA as an examinable subject in all schools by 2010; **Jamaica:** Establish healthy communities that are conducive for community members to be physically active; provide opportunities for children and youth to participate in supervised afterschool sports activities; establish polices, laws, and regulations supportive of a PA lifestyle, and supportive environment in schools, places of work, and communities; develop guidelines on physical education and sports for target group; include PA as a component of chronic disease management at government clinics; improve and evaluate facilities for engaging in PA in health services; design life skills program for schools, communities, and workplaces covering all aspects of healthy lifestyles, including PA; provide clean, safe, and green open space for all community members to participate in PA; **Brazil:** Promote population-wide PA; promote building of healthy urban spaces	**Bhutan:** Establish national standards for PA; establish a PA Act to regulate the built environment that supports active living; Ministry of Health will collaborate with Dratsang to integrate information and training sessions on PA; develop education materials for curricula aimed at encouraging PA in children, and provide supportive environments; **Sri Lanka:** Reactivate youth clubs, sports clubs, and young farmers club so as to promote PA; awareness program on PA for employees in institutions and also promotion of importance of PA through mass media	**Mongolia:** Develop and enforce training program of informal and distance learning on PA; develop population-specific PA guidelines and standards; provide advice for promotional measures of physical culture and active movement; create tax measures and market incentives directed towards promotion of PA; improve accessibility and quality of sports-related roads/areas and sports equipment/facilities and improve their safety lighting; **Viet Nam:** Develop physical exercise programs from preschool to undergraduate education; **Philippines:** Develop and implement health promotion activities for PA; regulate the built environment to promote PA; **Cambodia:** Develop presentation materials on PA; revise school curriculum for PA and promotion of women in sports; provide adequate sports facilities for school children and university students; collect data on availability of cycle ways and public parks; develop local strategies to promote PA; **China:** Actively create a sports and fitness environment; strengthen scientific guidance on mass sports activities; gradually increase the accessibility and utilization of various public sports facilities; enhance environment quality monitoring and evaluation; build a healthy environment and promote regular exercise
**Private sector**	NR	NR	**Turkey:** Increase the knowledge level regarding PA at the workplace; **Serbia:** Institutional organization of sport and recreative occasions, e.g., organize sport or recreative contests for workers or pensioners	**Jamaica:** Establish supportive networks and alliances with the private sector and create a partnership with the media to promote the value of PA; **Brazil:** Establish agreements with the productive sector to implement programs on PA, such as the Academia da Saude	**India:** Involvement of corporate sector to establish a sport culture	**Cambodia:** Involve sports personalities and media to promote PA
**National target for PA**	**Kenya:** Proportion of population that adopts a healthy diet and PA is 15% by 2016/2017	**Morocco:** 70% of the general population and 80% of children active by 2019	NR	**Cuba:** Increase proportion of adults doing PA to 40% and decrease prevalence of sedentary behavior in individuals ≥15 y to 32%; **Uruguay:** Average 30 min of moderate PA per day for adults and 1 h for adolescents and children; **Jamaica:** 40% increase in the number of persons having moderate levels of PA practiced for 30 min per day within 4 y; **Brazil:** Increase leisure-time PA levels	NR	**Malaysia:** Increase the proportion of people doing at least 30 min of PA per day, three times a week, compared to the First Malaysian Food Consumption Survey; **Philippines:** Reduction in prevalence of adults with high physical inactivity from 60.5% to 50.8%; **China:** Increase the proportion of the population with regular exercise to >32%

WHO classification of regions and countries was followed. Four of the countries (Mayotte, West Bank and Gaza, the Republic of Kosovo, and American Samoa) classified as LMICs by the World Bank in 2011 [25] were not WHO member states in 2011.

a Obtained from [107].

FYRM, the former Yugoslav Republic of Macedonia; NGO, nongovernmental organization; NR, not reported; PA, physical activity.

Four countries' policy documents (Georgia [57], Mongolia [69], Mauritius [63], and Chile [85]) contained detailed actions and elaborated an implementation plan for stakeholders. The need to develop sports infrastructure and urban planning (e.g., bicycle lanes and recreational centers) featured in the policy documents of Georgia [57], the Republic of Moldova [86], Turkey [62], and Mongolia [69], for instance. Five countries (Mauritius [63], Brazil [75], Samoa [80], the Republic of Moldova [86], and Serbia [87]) mentioned the need to promote physical activity among the elderly. Only four countries (Bhutan [66], the Philippines [73], Cuba [83], and the Republic of Moldova [86]) outlined specific strategies to address sedentary lifestyles, and five (Turkey [62], Cambodia [71], Jamaica [65], Serbia [87], and India [82]) documented explicit actions to involve the private sector in the promotion of physical activity.

## Discussion

Despite the global disease burden of NCDs in LMICs, policies that address at least one risk factor for NCDs were found in a minority of the LMICs reviewed, and only a handful of them comprehensively tackled NCDs through integrated action on various risk factors. Even if the 24 countries with unknown existence of a NCD prevention policy actually have such a policy, the proportion with countries tackling a risk factor would amount to 56% (78/140). This finding is discouraging, because in 2004, all countries expressed a strong commitment to action to address lifestyle, diet, and physical activity [20]. Our results show that, in spite of that official commitment, most LMICs are poorly prepared to tackle the NCD increase and that little progress has been made in recent years. This finding is consistent with the results of Alwan et al. [23], who reported the results of a survey in 2010 that was limited to countries with high NCD-related mortality.

Most of the policies in our review were poorly accessible and were only obtained after an extensive search or through personal contacts. Such a situation is certainly not favorable for benchmarking and communication of policies. In agreement with Sridhar et al. [88], we argue how better sharing of best practices and lessons learned with regard to policy development is needed to address the current NCD pandemic. Additional instruments and platforms to share lessons learned in policy development and implementation are needed. Policy databases with links to documents were created previously, but are restricted to nutrition action [89] or the European region [26]. An open-access, full-text global repository of initiatives and policies to address NCDs would be a great step forward. It could also contribute to global leadership and shared accountability in the global fight against NCDs, an issue that is long overdue [90]. Ideally, such a policy database would be connected to surveillance data on the main NCD risk factors, as suggested previously [23], and would facilitate tracking progress in the coming years. We are ready to organize such an open-access repository and invite interested policy makers to contact us for an update of the current database.

Priority setting and clear articulation of what needs to be done by stakeholders is a second key issue that emerged in this analysis. Countries seasoned in the fight against NCDs develop comprehensive strategies that focus on critical risk factors and what is expected of stakeholders [91]. In the present analysis, the level of detail and outlining of the organization of policy actions to undertake was generally discouraging. Only a minority of the policies reviewed surpassed description of policy actions and included a budget, implementation plan, time frame, and devolvement of responsibility for strategies to combat specific risk factors. Various policies describe strategies and actions for NCD prevention as “the need to develop and review dietary guidelines and recommendations for people suffering from nutrition-related NCDs” or use generic statements such as “create awareness of healthy eating lifestyle to control NCDs.” Such general statements are not informative, and clear actions need to be outlined in the policies to mobilize stakeholders for effective action [92].

Since its inception during the 1992 International Conference on Nutrition [93], the approach to streamline nutrition action in national policies has had limited success, partly because of the lack of strong leadership and commitment to lead concerted action involving various stakeholders [94]. The current scientific evidence and international experience in the fight against NCDs consistently indicates the need for comprehensive and integrated action on various risk factors [95]. Mobilization of the main actors—in particular, governments, international agencies, the private sector, civil society, health professionals, and individuals—is imperative [96]. An important limitation of most policies included in the analysis is the absence of plans, mechanisms, and incentives to foster multi-stakeholder and cross-sector collaboration. The food and nonalcoholic beverage industry, for instance, can play a role in the promotion of healthier lifestyles. However, before engaging with the private sector, government agencies should be aware of the need to manage potential conflicts of interest between the government and the private sector and should try to address these by defining clear roles, responsibilities, and targets to be achieved as a result of their collaboration [97]. Most strategies encountered in the policies were directed towards government agencies and consumers, and few were targeted at the business community, international agencies, or civil society. The United Nations Political Declaration on NCDs makes a strong call for multi-stakeholder partnerships to be leveraged for effective prevention of NCDs. Policy makers in LMICs may need additional support for the development of multi-stakeholder collaborations to address the burden imposed by NCDs as well as their root causes.

In our review of governmental policies relating to NCD prevention in LMICs, strategies to increase fruit and vegetable intake were the most frequent dietary action for NCD prevention. This is hardly surprising, as fruit and vegetable interventions were taken up early on in LMICs, primarily to address prevailing micronutrient deficiencies such as vitamin A deficiency [98]. Many of these experiences, however, are restricted to the development of food-based dietary guidelines or incentives targeted towards the agricultural sector. Policy measures to achieve better diet will require constructively engaging much more with a wider range of stakeholders, in particular the food industry, retail, and the catering sector [99]. The difficulty of developing a comprehensive policy response and integrated package of strategies is not restricted to NCDs alone, and has previously been observed in an in-depth analysis of high-burden countries for child malnutrition [100]. We also note that various countries have developed strategies to reduce total fat intake, despite convincing evidence that it is the reduction of saturated and trans-fatty acids in particular, and not total fat intake, that is effective to address NCDs [101].

Most strategies encountered in the policy documents focused on consumers and aimed to prevent NCDs through awareness creation, education (i.e., labeling), or changing individuals' behavior. The traditional approach to addressing lifestyle changes in individuals has met with very limited success. It is widely accepted that the environmental context drives individual diets and lifestyle [102] and that programs need to incorporate environmental determinants (i.e., the quantity, quality, or price of dietary choices, or the built environment for physical activity) in order to be effective. Such policy measures, in particular those addressing the private sector, were poorly elaborated in the policy documents [103].

A key issue is the actual implementation of policy measures in relation to what was articulated in the documents. The findings of this review indicate that few LMICs have made significant steps in the development of a comprehensive set of strategies to address NCDs. Although an in-depth evaluation of actual implementation, effects, and resources allocated has not been opportune to date, we hope that our findings provide baseline data and encourage countries to develop monitoring and evaluation mechanisms to assess policy response in due time. Documenting the effectiveness of population-based NCD prevention policies will be a critical factor of success to ensure effective action in LMICs [4].

For this review, we were able to assess documents in all languages received. Because of language constraints, however, two of the documents [74],[87] were coded by only one researcher. To assess the content of the policy of Iran, we relied on translations by experienced senior Iranian researchers. All other policy documents were obtained in Spanish, Portuguese, French, or English and were analyzed accordingly by the research team. For China and the Russian Federation, appropriate English versions of the policies were obtained from the Chinese Centers for Disease Control and the United States Department of Agriculture, respectively. Despite indications of availability of relevant policies in the European region [26], language limitations did not allow us to search the websites of a number of countries such as Azerbaijan, Belarus, and the Russian Federation.

The restriction of our review to only national policies presents a number of limitations. The mere presence or absence of policies or strategies for NCDs in a policy document does not necessarily reflect concrete action. Conversely, nutritional interventions have been implemented in some countries without a policy being developed and published [104]. In addition, this review assessed the contents of the policy documents as they were published and did not capture local or regional activities, or initiatives that emerged after the publication of the policies. The findings from a survey in countries with a high burden of NCDs, such as Thailand and South Africa, illustrate this discrepancy [23]. The contents might have been modified over time in response to new scientific findings, emerging nutritional challenges, or changes in the countries' priorities [91]. In addition, it is important to point out that we extracted only actions that explicitly referred to one of the risk factors analyzed. Generic statements such as “development of food-based dietary guidelines” or “establishment of fiscal measures for a healthy diet” were hence not coded.

The present review shows that the policy response to address current NCD challenges through diet and physical inactivity in LMICs is inadequate since endorsement of the Global Strategy on Diet, Physical Activity and Health [20]. LMICs urgently need to scale up interventions and develop integrated policies that address various risk factors for NCD prevention through multi-stakeholder collaboration and cross-sector involvement. Clear and prioritized actions are needed to harness the NCD epidemic. Such actions need to be documented in policy documents that are publicly available to share lessons learned, promote engagement with the stakeholders, and stimulate accountability and leadership in the fight against the burden of NCDs in LMICs. The establishment of an open-access and publicly accessible database of policy documents with regular systematic reviews of policy development might prove to be an incentive in this regard.

## Supporting Information

Alternative Language Abstract S1 **Portuguese translation of the abstract by VC.**
(DOCX)

Alternative Language Abstract S2 **Spanish translation of the abstract by FMAS.**
(DOCX)

Alternative Language Abstract S3 **French translation of the abstract by DR.**
(DOCX)

Table S1 **Availability of national policy documents and strategies for noncommunicable disease prevention in low- and middle- income countries by WHO region.**
(XLSX)

Text S1 **Original review protocol.**
(PDF)

Text S2 **PRISMA checklist of the review.**
(PDF)

## Abbreviations

NCD: noncommunicable disease
LMICs: low- and middle-income countries
WHO: World Health Organization
