# Supplementary material for: Diet and Physical Activity for the Prevention of Noncommunicable Diseases in Low- and Middle-Income Countries: A Systematic Policy Review
Source: PLoS Med. 2013 Jun 11;10(6):e1001465. doi: 10.1371/journal.pmed.1001465 (PMC3679005; doi:10.1371/journal.pmed.1001465)
Supplement: Alternative Language Abstract S1 — Portuguese translation of the abstract by VC. (DOCX) [file pmed.1001465.s001.docx]

**Supporting Information: Translation of the abstract Diet and Physical Activity for the Prevention of Non Communicable Diseases in Low and Middle-Income Countries: A Systematic Policy Review into Portuguese by author Vanessa Candeias**

**Alimentaçao e Actividade Fisica para a Prevenção de Doenças Crónicas Não Transmissíveis em Países de Médio e Baixo Rendimento: análise sistemática de políticas**

Contexto

A prevalência de doenças crónicas não transmissiveis (DCNT) está a aumentar rapidamente em países de médio e baixo rendimento e constituem uma importante causa de mortalidade. Apesar de uma chamada global para a acção estar a ocorrer há já vários anos, o progresso no desenvolvimento de políticas para as DCNT não está documentado. Esta análise sistemática das estratégias para prevenir DCNT em países de médio e baixo rendimento apresenta uma referência de base para a monitorização da resposta para o desenvolvimento destas políticas.

Métodos e resultados

Os autores analisaram como políticas em países de médio e baixo rendimento descrevem ações para lidar com o consumo de sal, gorduras e hortofrutícolas e a prática de actividade física. Foi conduzida uma análise estruturada do conteúdo das políticas de nutrição, DCNT e saúde publicadas entre Janeiro de 2004 e Janeiro de 2013 dos 140 Estados Membros da OMS. Avaliou-se a disponibilidade destas políticas em 83% (116/140) dos países. Estratégias para as DCNT foram encontradas em 47% (54/116) dos países de médio e baixo rendimento incluídos, mas apenas uma minoria dos documentos incluía acções para a promoção de alimentação saudável e actividade física. O número de políticas que incluía nos seus objectivos pelo menos um dos factores de risco, comparado com outras regiões, era mais baixo nas regiões das Américas, África e Mediterraneo Oriental. Dos países incluídos na análise, apenas 12% (14/116) têm uma política que visa os quatro fatores de risco e 25% dos países (29/116) inclui apenas um dos fatores de risco. Estratégias visando o setor privado foram menos frequentes em comparação com estratégias dirigidas à população em geral ou aos legisladores.

Conclusões

Esta análise indica uma incoerência entre o peso das DCNT e a resposta em termos de políticas ao nível nacional. Os órgãos responsáveis precisam urgentemente de desenvolver políticas abrangentes e inclusivas que visem melhorar a alimentação e actividade física ao nível nacional.
