# Supplementary material for: Diet and Physical Activity for the Prevention of Noncommunicable Diseases in Low- and Middle-Income Countries: A Systematic Policy Review
Source: PLoS Med. 2013 Jun 11;10(6):e1001465. doi: 10.1371/journal.pmed.1001465 (PMC3679005; doi:10.1371/journal.pmed.1001465)
Supplement: Alternative Language Abstract S2 — Spanish translation of the abstract by FMAS. (DOCX) [file pmed.1001465.s002.docx]

**Supporting Information: Translation of the abstract Diet and Physical Activity for the Prevention of Non Communicable Diseases in Low and Middle-Income Countries: A Systematic Policy Review into Spanish by author Florencia Maria Aguirre Seret**

**La dieta y la actividad física para la prevención de la Enfermedades no Transmisibles en Países de ingresos medios y bajos: Una revisión sistemática de políticas**

Introducción

Las enfermedades no transmisibles (ENT) relacionadas con la dieta están aumentando rápidamente en los Países de ingresos medios y bajos (PIMB) y constituyen la principal causa de mortalidad. A pesar de que el llamado a la acción global ha estado resonando durante años, los avances en el desarrollo de políticas en los PIMB no se han documentado. Esta revisión de las estrategias de prevención de las ENT en los PIMB proporciona un punto de referencia con la cual pueden ser comparadas las respuestas políticas a lo largo del tiempo.

Métodos y resultados

Exploramos cómo las políticas en PIMB destacan las acciones para la ingesta de sal y el consumo de grasas, frutas y verduras y la actividad física como factores de riesgo de enfermedades no transmisibles. Se llevó a cabo un análisis estructurado del contenido de las políticas nacionales de nutrición, de las enfermedades no transmisibles y de salud publicadas entre enero de 2004 y enero de 2013 de 140 PIMB miembro de la OMS. Se evaluó la disponibilidad de las políticas en el 83% (116/140) de los países. Estrategias para las ENT fueron encontradas en el 47% (54/116) de los PIMB investigados pero sólo una minoría propuso medidas para promover dietas más saludables y la actividad física. La cobertura de las políticas dirigidas específicamente al menos uno de los factores de riesgo analizados fue más baja en África, Europa, América y la región del Mediterráneo Oriental en comparación con otras regiones. De los países estudiados, sólo el 12% (14/116) propone una política que aborde todos los cuatro factores de riesgo y el 25% (29/116) aborda sólo uno de los factores de riesgo analizados. Estrategias dirigidas al sector privado fueron menos frecuentes en comparación con aquellas dirigidas hacia el público en general o hacia los responsables de la formulación de políticas.

Conclusión
Esta revisión indica la desconexión entre la carga de las enfermedades no transmisibles y la respuesta de las políticas nacionales en los PIMB. Los responsables de formular políticas necesitan con urgencia desarrollar políticas integrales y de múltiples partes interesadas ​​para mejorar la calidad de la dieta y la actividad física.
