# Supplementary material for: Diet and Physical Activity for the Prevention of Noncommunicable Diseases in Low- and Middle-Income Countries: A Systematic Policy Review
Source: PLoS Med. 2013 Jun 11;10(6):e1001465. doi: 10.1371/journal.pmed.1001465 (PMC3679005; doi:10.1371/journal.pmed.1001465)
Supplement: Alternative Language Abstract S3 — French translation of the abstract by DR. (DOCX) [file pmed.1001465.s003.docx]

**Supporting Information: Translation of the abstract Diet and Physical Activity for the Prevention of Non Communicable Diseases in Low and Middle-Income Countries: A Systematic Policy Review into French by author Dominique Roberfroid**

**Alimentation et exercice physique pour la prévention des maladies non transmissibles dans les pays à faible et moyen revenu: une revue systématique des politiques**

Contexte

Les maladies non transmissibles (MNT) liées à l’alimentation augmentent rapidement dans les pays à faible et moyen revenu (PFMR) et constituent une cause majeure de mortalité. Bien que depuis des années le développement d’une stratégie globale est souhaité, les progrès dans la mise en place de politiques adéquates dans les PFMR n’ont pas encore été documentés. Notre revue des politiques de prévention des MNT dans les PFMR fournit un jalon pour mesurer la réponse politique à l’avenir.

Méthodologie et résultats

Nous avons passé en revue comment les politiques dans les PFMR mettent en exergue des actions visant à diminuer la consommation de sel et de graisse, et à promouvoir la consommation de fruits et de légumes ainsi que l’exercice physique. Nous avons procédé à une analyse structurée de contenu des politiques concernant la nutrition, les MNT et la santé publiées entre janvier 2004 et janvier 2013 dans 140 PFMR membres de l’OMS. Nous avons observé l’existence d’ une telle politique dans 83% (116/140) des PFMR. Une stratégie pour prévenir les MNT était décrite dans 47% (54/116) des PFMR inclus, mais seulement une minorité proposait des actions de promotion d’une alimentation plus saine et de l’exercice physique. L’existence d’une politique visant spécifiquement au moins un des facteurs de risque mentionnés plus haut était moins fréquente en Afrique, en Europe, en Amérique et dans la région Est de la Méditérranée que dans les autres régions. Seulement 12% (14/116) des pays analysés présentaient une politique concernant les quatre facteurs de risque, et 25% (29/116) avaient une politique concernant un seul de ces facteurs. Les stratégies visant le secteur privé étaient moins fréquentes que celles visant le public en général ou les preneurs de décision.

Conclusions

Cette revue souligne la déconnection entre la charge morbide des MNT et la réponse politique au niveau national dans les PFMR. Les preneurs de décision doivent rapidement mettre en place des politiques globales et multi-sectorielles pour améliorer la qualité de la consommation alimentaire et promouvoir l’exercice physique.
