# Supplementary material for: Diet and Physical Activity for the Prevention of Noncommunicable Diseases in Low- and Middle-Income Countries: A Systematic Policy Review
Source: PLoS Med. 2013 Jun 11;10(6):e1001465. doi: 10.1371/journal.pmed.1001465 (PMC3679005; doi:10.1371/journal.pmed.1001465)
Supplement: Text S1 — Original review protocol. (PDF) [file pmed.1001465.s005.pdf]

# Nutrition policy in developing countries: where do we stand?

A global documentary analysis of national policy plans

## Introduction and background

Disease patterns are changing globally and developing countries are no longer facing only traditional infectious diseases and predominantly undernutrition. Diet related chronic diseases are increasingly rapidly worldwide and various countries currently face both infectious as well as chronic diseases. This puts a considerable burden on the health system and other implicated sectors and actions are needed to curb the current trend.

WHO's Global Strategy on Diet, Physical Activity and Health was an international milestone in global political commitment to act effectively. Amongst others, the resolution urged member states to

> *" to develop, implement and evaluate actions recommended in the strategy, as appropriate to national circumstances and as part of their overall policies and programmes, that promote individual and community health through healthy diet and physical activity, and reduce the risks and incidence of noncommunicable diseases;*
>
> *(...)*
> *to strengthen existing, or establish new, structures for implementing the strategy through the health and other concerned sectors, for monitoring and evaluating its effectiveness and for guiding resource investment and management to reduce the prevalence of non-communicable diseases and the risks related to unhealthy diet and physical inactivity;*
>
> *to define for this purpose, consistent with national circumstances: national goals and objectives"*

The WHO office for Europe has launched its second action plan for food and nutrition policy. It contains guidelines for member states to develop comprehensive policies thereby using interdisciplinary partnerships for effective action. It is unclear what the

options are for such guidelines on an international level. A global documentary analysis of national nutrition action plans will be undertaken to shed light on this.

## Objective

The main aim of this study is to evaluate the progress done, after 2004, in the development and publication of national nutrition/diet and non-communicable diseases policies of the Member States (MS) classified as World Bank low and lower-middle income countries (LMIC).

**Objectives:**

1) To assess how many Member States have a national diet/nutrition, NCD prevention or health policy published after 2004.
2) To analyze the content of the existing policies considering the recommendations for Member States specified in the WHO Global Strategy on Diet, Physical Activity and Health (DPAS) in particular those related to diet (fruit and vegetable, salt and fat) and physical activity.

**Methodology**

**Design:**

A qualitative analysis of publicly available nutrition, NCD or health policies, published after 2004, will be conducted to evaluate whether they include or not recommendations as outlined by DPAS. An internet search will be conducted be conducted for all LMIC Member States. The internet search was conducted using the Google engine search to retrieve the policy documents from the following MS websites:

1. Ministry of Health
2. Ministry of Agriculture
3. Ministry of Education
4. Ministry of Youth
5. Government portals

6. Nutrition societies

For those MS for which no policy was retrieved through the web search, an official email requesting the official policy documents that were relevant for this study will be sent by Ghent University to respective parties of those Member States. The respective parties in this context will be:

1. Ministry of Health
2. Ministry of Agriculture
3. Ministry of Education
4. Ministry of Youth
5. Nutrition societies

The same request email will need also to be sent by WHO to respective WHO Regional Offices. If no official policy document was found after the performance of the steps described above, the MS was registered as not having publicly available policies.

**Inclusion criteria:**

The following inclusion criteria must be met for any of the collected documents to be included in the analysis:

- Official approved policy of a WHO Member State classified as low and lower-middle income countries (according to the World Bank classification: http://web.worldbank.org/WBSITE/EXTERNAL/DATASTATISTICS/0,,contentMDK:20420458~menuPK:64133156~pagePK:64133150~piPK:64133175~theSitePK:239419,00.html).
- Publicly available document, published after 2004.
- Be related to diet/nutrition and/or primary prevention of non-communicable diseases through national nutrition policies (NP), national NCD policies (NCDP), or national health policies (HP).
- Published in English, French, Portuguese or Spanish.

The definitions of policy/strategy/action plan and programme vary in the literature and among the national documents. In this project report all national documents that included

the objectives and guidelines for action in the domain of diet and/or prevention of non-communicable diseases will be included in the analysis.

All the nutrition, NCD and health policies publicly available were retrieved and saved for all LMIC MS. (Note: this is an amendment to the previous version that contained a step wised method of selection of policy documents. After a first assessment, it was decided to include all eligible document for the review rather than a selection based on name or title of the document.)

We will explore Policy documents related to following Key words

- Health
- Nutrition (incl obesity, physical activity, NCD and overweight)
- Food and food security

Consequently, we will not review policy documents for poverty reduction, PRSP, ..

**Organization**

A team of students will work on a particular list of countries (Annex 2) . The division of the list of countries will follow that of WHO[1]. The WHO is operational through its regional offices. There are 6 regional offices, namely WHO African Region , WHO Region of the Americas, WHO South-East Asia Region, WHO European Region , WHO Eastern Mediterranean Region and the WHO Western Pacific Region.

**Methods for comparative analysis**

MS policies eligible for the study will be analyzed as follows:

1. **Data coding**

Coding or categorizing data plays an important role in analysis as it organizes the data by subdividing and assigning categories to the data. The policy documents will each be coded by more than one researcher, in order to minimize the possibility of subjective

[1] http://www.who.int/about/structure/en/

coding results. For this study, the contents of the eligible policy documents will be coded in two phases:

- Phase 1: Keyword coding

Policy documents will be scanned for pre-identified keywords that are relevant to the study objectives (Table 1). The keywords were built based on the elements that are deemed to be important in the framework of a policy document with focus on the promotion of a healthy nutrition in the context of non-communicable diseases prevention and considering the recommendations to Member States included in the DPAS. The keywords used for coding were translated into each of the four languages used for the analysis (English, French, Spanish, Portuguese). The coding is done using NVivo (qualitative data analysis computer software) and the search queries were made separately for each language within each of the WHO regions (Table 1.).

The coded text is saved in NVivo as tree nodes. These nodes are named with the English keywords but they contain all the text that correspond to the same keywords in any of the languages.

**Table 1. Guideline for keywords used for phase 1 coding**

| Language | Keyword | Nvivo query |
|---|---|---|
| English | Agricultural | agricultural |
| | Breastfeeding | breastfeeding |
| | Fat | fat |
| | Fiscal | fiscal |
| | Food labeling | food OR label OR labeling |
| | Food marketing | marketing |
| | Fruits and vegetables | fruit OR vegetable |
| | Iodization | Iodization |
| | Physical activity/exercise | physical OR exercise |
| | Salt | salt |

| | | |
|---|---|---|
| | Sugars | sugar |
| | Trans-fatty acids | trans OR fatty OR acids |
| | | |
| Spanish | Acidos grasos trans | ácidos OR grasos OR trans |
| | Actividad física/ejercicio | física OR ejercicio |
| | Agricultura | agricultura |
| | Azúcar/azucares | azucar OR azúcar |
| | Comercialización | comercialización OR publicidad |
| | Etiquetado | Etiquetado OR etiqueta |
| | Fiscal | fiscal |
| | Frutas y verduras | Frutas OR verduras OR hortalizas OR legumbres |
| | Grasas | grasa |
| | iodización | Iodización OR yodada OR yodo |
| | Lactancia materna exclusiva | lactancia OR materna |
| | Sal | sal |
| | | |
| French | acides gras trans | |
| | Commercialisation, publicité | |
| | étiquetage | |
| | exercice physique | |
| | Fiscal | |
| | Fruits et légumes | |
| | graisses | |
| | l'agriculture | |
| | l'allaitement maternel exclusif | |

| | | |
|---|---|---|
| | Sel | |
| | sel iodé | |
| | sucres | |
| | | |
| Portuguese | A rotulagem de alimentos | |
| | Açúcares | |
| | Agrícola | |
| | Amamentação | |
| | Atividade Física,_exercício | |
| | Comercialização de alimentos | |
| | Fiscal | |
| | Frutas e legumes | |
| | Gorduras | |
| | Iodização | |
| | Os ácidos gordos trans | |
| | Sal | |
| | | |
| | | |

In addition, each of the activities will be coded for following target groups

| |
|---|
| • General public and consumers |
| • Government |
| • Private sector |
| • National salt intake target |

- Phase 2: Assessment of text coded in phase 1

After coding the documents in phase one, the text retrieved in the process was assessed to determine the context in which the keyword was being used and whether or not it corresponded to a DPAS component within the policy.

Initially the text was coded using the NVivo free nodes in order to identify and name the different components of the policy in which the keywords were found. These free nodes were then merged into the following tree nodes:

- **Background:** the text gives an introduction, context or background for the policy.
- **Axis:** the text describes the principles that determined the content of the policy.
- **Objectives:** the text states the aims, goals, vision?, expected outcomes? and objectives of the policy.
- **Strategies:** the text describes the different strategies described in the policy in order to achieve the DPAS recommendations. Actions, participating stakeholders, and law *production* are included in this category.
- **Other context:** text was included here when the keywords were used in a context other than the DPAS recommendations.

**2. Mapping of policy availability of the Member States**

This step is related with study objective number 1. For this purpose a new consolidated policy database containing all the eligible policy documents will be constructed and a map will be produced. This map will provide an overview of availability of a national policy on nutrition/diet and/or non-communicable diseases prevention published by WHO Member States.

**3. Comparison and analysis of eligible policy document with DPAS**

This step is related with study objective number 2. A table (as per table 2) will be constructed to assist the comparison and analysis purposes. This table will be constructed with the data sourced out from the coded eligible documents.

**Table 2. Member states policy content vs. DPAS (which is represented by the selected keywords)**

| Member states | DPAS recommendation (represented by the selected keywords) | | | | | | |
|---|---|---|---|---|---|---|---|
| | Category: Setting | | | | | Category: Operational | |
| | Workplace | School | Community | Urban | Rural | Funding | Human resources |
| A | | | | | | | |
| B | | | | | | | |
| C | | | | | | | |
| D | | | | | | | |
| E | | | | | | | |

Note: the cells highlighted in yellow to be filled with "Y" if the content of the Member State policy is in line with recommendation or with "N" if it is not in line

**Ethical considerations**

There are no ethical concerns to be considered as all information used for this report will be in the public domain, and neither human subjects nor unpublished data will be used.

To acquire clearance from the Member States for using their policy documents for this study, the request for the clearance will be included in the email sent to those Member States where the policy documents was not obtained (Member States with missing policy documents).

The manuscript will be sent for clearance to WHO and IDF before publication.

## Contacts

**Contacts at WHO,**
**Chronic Diseases and Health Promotion Department:**

Godfrey Xuereb (xuerebg@who.int )
Team Leader, Global Strategy for Diet, Physical Activity and Health Surveillance and Population-based Prevention Unit

Vanessa Candeias (candeiasv@who.int )
Surveillance and Population-based Prevention Unit

Diana Syafitri (Syafitrid@who.int )
Surveillance and Population-based Prevention Unit

**Contacts in Ghent University/institute of Tropical Medicine**

Carl Lachat (CLachat@itg.be )
(coordinator, affiliation see below Patrick Kolsteren)

Patrick Kolsteren (PI)
pkolsteren@itg.be

Head Nutrition and Child Health Unit
Department of Public Health Institute of Tropical Medicine Belgium

Department of Food Safety and Food Quality, Faculty of Bioscience Engineering, Ghent University, Belgium

## Timing

The review started in December 2008 and was completed in October 2011.

Annex: Template of email

---

To: Mr(s)
title
Country X

Dear Mrs. Andrea Robin

We are an interdisciplinary task force that currently is carrying out a study on nutrition policy. The study is coordinated by the Nutrition and Child Health Unit of the Prince Leopold Institute of Tropical Medicine and the Department of Food Safety and Food Quality Research Group Food Chemistry and Human Nutrition of the Ghent University, Belgium. The objective of this study is to review nutrition policy documents in developing countries. The study is the continuation of the following publications:

*Lachat C, Roberfroid D, Huybregts L, Van Camp J., Kolsteren P. (2008) Incorporating the catering sector in nutrition policies of WHO European region: is there a good recipe?*

*C. Lachat, J. Van Camp, S. De Henauw, C. Matthys, Y. Larondelle, A.M. Remaut- De Winter & P. Kolsteren (2005). A concise overview of national nutrition action plans in the European Member States. Journal of Public Health Nutrition, 8 (3) 266-274*

Hereby I contact you to ask a kind favour if you could help to provide us with the:

1. National nutrition policies of country X

If you have a question regarding this email, please do not hesitate to contact me.

Many thanks for your assistance.

Best regards,

Carl Lachat, coordinator
Name of the researcher for Latin America and the Caribbean
